# Characterization of MicroRNA-200 pathway in ovarian cancer and serous intraepithelial carcinoma of fallopian tube

**DOI:** 10.1186/s12885-017-3417-z

**Published:** 2017-06-17

**Authors:** Junzheng Yang, Yilan Zhou, Shu-Kay Ng, Kuan-Chun Huang, Xiaoyan Ni, Pui-Wah Choi, Kathleen Hasselblatt, Michael G. Muto, William R. Welch, Ross S. Berkowitz, Shu-Wing Ng

**Affiliations:** 1 Department of Obstetrics/Gynecology and Reproductive Biology, Brigham and Women’s Hospital, Boston, MA 02115 USA; 2 School of Medicine and Menzies Health Institute Queensland, Griffith University, QLD, Nathan, 4111 Australia; 3 Department of Pathology, Brigham and Women’s Hospital, Boston, MA 02115 USA; 4 Laboratory of Gynecologic Oncology, Brigham and Women’s Hospital, 221 Longwood Avenue, Boston, MA 02115 USA

**Keywords:** MicroRNA, Expression analysis, Ovarian tumors, Fallopian tube tumors

## Abstract

**Background:**

Ovarian cancer is the leading cause of death among gynecologic diseases in Western countries. We have previously identified a miR-200-E-cadherin axis that plays an important role in ovarian inclusion cyst formation and tumor invasion. The purpose of this study was to determine if the miR-200 pathway is involved in the early stages of ovarian cancer pathogenesis by studying the expression levels of the pathway components in a panel of clinical ovarian tissues, and fallopian tube tissues harboring serous tubal intraepithelial carcinomas (STICs), a suggested precursor lesion for high-grade serous tumors.

**Methods:**

RNA prepared from ovarian and fallopian tube epithelial and stromal fibroblasts was subjected to quantitative real-time reverse-transcription polymerase chain reaction (qRT-PCR) to determine the expression of miR-200 families, target and effector genes and analyzed for clinical association. The effects of exogenous miR-200 on marker expression in normal cells were determined by qRT-PCR and fluorescence imaging after transfection of miR-200 precursors.

**Results:**

Ovarian epithelial tumor cells showed concurrent up-regulation of miR-200, down-regulation of the four target genes (*ZEB1, ZEB2, TGFβ1* and *TGFβ2*), and up-regulation of effector genes that were negatively regulated by the target genes. STIC tumor cells showed a similar trend of expression patterns, although the effects did not reach significance because of small sample sizes. Transfection of synthetic miR-200 precursors into normal ovarian surface epithelial (OSE) and fallopian tube epithelial (FTE) cells confirmed reduced expression of the target genes and elevated levels of the effector genes *CDH1, CRB3* and *EpCAM* in both normal OSE and FTE cells. However, only FTE cells had a specific induction of CA125 after miR-200 precursor transfection.

**Conclusions:**

The activation of the miR-200 pathway may be an early event that renders the OSE and FTE cells more susceptible to oncogenic mutations and histologic differentiation. As high-grade serous ovarian carcinomas (HGSOC) usually express high levels of CA125, the induction of CA125 expression in FTE cells by miR-200 precursor transfection is consistent with the notion that HGSOC has an origin in the distal fallopian tube.

**Electronic supplementary material:**

The online version of this article (doi:10.1186/s12885-017-3417-z) contains supplementary material, which is available to authorized users.

## Background

Ovarian cancer is the leading cause of death among gynecologic diseases in Western countries. Ovarian tumors of epithelial origin can be classified as benign, low malignant potential (borderline), or malignant according to the World Health Organization criteria [1]. Because of the lack of sensitive tests for the detection of early stage of the disease, which often lacks obvious clinical symptoms, patients with malignant epithelial ovarian tumors have a 5-year survival rate of about 30% [2]. In contrast, the survival rate will exceed 90% for five years if the disease is diagnosed in Stage I [3].

Ovarian cancer can be divided into four major histologic subtypes based on morphologic criteria corresponding to the different types of epithelia in the Müllerian ducts, the embryological structures that eventually form vagina, cervix, uterus and fallopian tubes [4–6]. The most common serous tumors comprise about 50% of primary epithelial ovarian tumors and consist of epithelial cells resembling those of the fallopian tube with high metastatic potential. The other three subtypes are mucinous, endometrioid, and clear cell ovarian carcinomas. The origin of ovarian cancer has been greatly debated. The incessant ovulation hypothesis, based on epidemiologic observations that women with a low number of pregnancies or infertility and, hence, a greater number of ovulatory cycles have an increased risk of developing ovarian cancer, has suggested that ovarian cancer develops from the surface epithelium and the associated cortical inclusion cysts [7, 8]. Recently, epidemiologic and molecular pathologic studies on *Brca1* and *Brca2* carriers have suggested that a disproportionate number of high-grade serous ovarian carcinomas (HGSOC) originate in the distal fallopian tube, initiating as serous tubal intraepithelial carcinoma (STIC) [9–11]. *p53* mutations and expression of γ-H2AX, which are evidence of DNA damage frequently observed in high-grade pelvic serous carcinomas, also appeared in STIC and foci of benign tubal mucosa in continuity with STIC [10]. A study on serous ovarian carcinomas without *Brca* mutations have shown that approximately one-half of these tumors co-existed with a STIC, and *p53* mutation analysis in the STIC and metastatic tumors disclosed the same mutations, suggesting that they were genetically linked [12]. These emerging data offer evidence that a significant percentage of familial and sporadic HGSOC could be explained by an origin in the distal fallopian tube.

MicroRNAs (miRNAs) are a family of small (~22 nucleotides) noncoding RNA molecules that are evolutionarily conserved and are expressed in a tissue-specific and developmental stage-specific manner [13]. There is growing evidence to support the notion that many miRNAs can potentially target different mRNAs [14] and are master regulators in development [15, 16] and other pathologic processes including cancers. 50% of miRNA genes are frequently located at fragile chromosomal sites and genomic regions involved in cancers [17, 18]. The expression profiles of miRNAs can classify tumor types [19] and are associated with tumor diagnosis and prognosis [20]. MiRNAs can function as oncogenes or tumor suppressors [21, 22] and a recent study has shown that changes of miRNA expression are causal, rather than consequential, of cellular transformation [23]. Hence, miRNAs have great potential to be developed as a novel class of disease diagnostics and therapeutic targets [24, 25].

In an in vitro three-dimensional (3D) culture study to explore the molecular changes involved in inclusion cyst formation and tumor invasion, we have identified concurrent elevated expression of miR-200 family members (miR-200a, miR-200b, miR-200c, miR-141, and miR-429) and the effector protein E-cadherin (CDH1) in the cancer 3D cultures compared to the 3D cultures derived from normal human ovarian surface epithelial (OSE) cells [26]. Suppression of CDH1 expression in ovarian cancer cells disrupted inclusion cyst formation and collective movement demonstrated in the cancer 3D cultures [26]. MiR-200 family and miR-205 are the major regulators of epithelial-mesenchymal transition (EMT) and cell differentiation by suppressing the transcriptional repressors *ZEB1* and *ZEB2* [27] and transforming growth factor β (*TGFβ*) [28]. In this report, we describe an extensive expression and clinical association analysis of the miR-200 pathway in a panel of clinical ovarian and fallopian tube samples. Functional studies suggest the involvement of miR-200 in CA125 production specifically in fallopian tube epithelial (FTE) cells.

## Methods

### Cell lines, tissues, and primary cultures

Ovarian cancer cell lines DOV13, CAOV3, OVCA420, OVCA429, OVCA432, OVCA680, and OVCA810 were established in our laboratory from ovarian carcinomas obtained from different patients. SKOV3 was obtained from American Tissue Culture Collection (ATCC). RMG-1 was purchased from Japanese Collection of Research Bioresources (JCRB Cell Bank). All ovarian cancer cell lines except RMG-1 and OCA810 were grown in medium 199 (Sigma-Aldrich, St. Louis, MO) and MCDB 105 (Sigma-Aldrich, St. Louis, MO) (1:1) supplemented with 10% fetal calf serum (FCS). RMG-1 and OVCA810 were cultured with DMEM-F12 HAM medium (Life Technologies, Grand Island, NY) plus 10% FCS. Ovarian surface epithelial (OSE) and fallopian tube epithelial (FTE) primary cultures were derived from ovarian and distal fallopian tube scrapes from patients who underwent prophylactic salpingo-oophorectomies. Ovarian and fallopian tube specimens were collected with an IRB approved protocol at Brigham and Women’s Hospital in Boston.

### Laser-capture microdissection, RNA extraction, and quantitative real-time reverse-transcription PCR (qRT-PCR)

A Leica CM3050S cryostat was used to cut 7 μm-thick sections from frozen tissues embedded in OCT compound. The sections were placed on a polyethylene terephthalate (PET) UV-absorbing membrane-slide (Leica Microsystems), fixed using absolute methanol, and then stained with Hematoxylin to reveal tissue morphologies. A Leica AS LMD laser microdissection system (Leica Microsystems) was used to procure pure cell populations from the tissue sections and the RNA was extracted using an RNAqueous kit (Life Technologies, Carlsbad, CA). TRIzol reagent (Life Technologies, Carlsbad, CA) was used to extract RNA from primary cultures. TaqMan MicroRNA Reverse Transcription Kit and TaqMan MicroRNA assay kits (Applied Biosystems, Foster City, CA) were used for qRT-PCR determination of miRNA levels, performed on a 7300 Real-Time PCR System (Applied Biosystems, Foster City, CA). *RNU6B* RNA was used as the internal control to normalize sample input. For target gene and effector gene determination, High Capacity cDNA Reverse Transcription Kit (Applied Biosystems, Foster City, CA) was used for cDNA synthesis. The synthesized cDNAs were adjusted in 1× TaqMan PreAmp Master Mix (Applied Biosystems, Foster City, CA), mixed with a pool of TaqMan Gene Expression Assays for target and effector genes, and a 14-cycle multiplexed pre-amplification PCR reaction was performed. The pre-amplified cDNAs and individual TaqMan Gene Expression Assays were loaded to individual wells of a BioMark 48.48 Dynamic Array (Fluidigm Corporation, South San Francisco, CA), which allowed simultaneous run of 48 qPCRs for 48 samples on a BioMark System (Fluidigm Corporation, South San Francisco, CA). Data collection and analysis was performed automatically by the Fluidigm instrument. Gene expression levels were determined using 2 ^-ΔΔCT^ method [29]. The data were exported to TIGR MEV (MultiExperiment Viewer) V4.0 (http://www.tm4.org) for heat map construction.

### Precursor transfection assay

Normal OSE and FTE cells were seeded at 2 × 10^5^/well in a 6-well culture plate (BD Biosciences, San Jose, CA). Ambion Pre-miR miRNA precursors for miR-200 family members and miR-205, as well as the nontarget negative control precursor, were purchased from Life Technologies (Carlsbad, CA). Transfection of the precursors into the cells was performed using the siPORT *NeoFX* transfection agent (Life Technologies, Carlsbad, CA) according to the manufacturer manual. The cells were harvested after 2 days and RNA was extracted for qRT-PCR.

### Immunofluorescence microscopy and Western blot analysis

FTE cells were seeded at 1 × 10^4^/well in an 8-well chamberslide (BD Biosciences, San Jose, CA). Cells were transfected with the miRNA precursors on the second day, using siPORT *NeoFX* transfection agent as described above. After 4 days of incubation, cells were fixed in 4% paraformaldehyde (Sigma-Aldrich, St. Louis, MO) and permeabilized with PBS containing 0.5% Triton X-100 (Sigma-Aldrich, St. Louis, MO). After blocking with 10% FBS, mouse monoclonal antibodies for CA125 (Thermo Fisher Scientific, Waltham, MA) and EpCAM (Dako North America, Inc. Carpinteria, CA) were added and incubated at room temperature for 2 h, then with Alexa Fluor 647 conjugated secondary antibodies (Invitrogen, Carlsbad, CA). A mounting medium with DAPI (Vector Laboratories, Burlingame, CA) was used for counterstaining. Microscopic images were captured by a Leica DM IRE2 fluorescence microscope (Leica Microsystems, Bannockburn, IL) and analyzed by the OpenLab Cell Imaging System software (Leica Microsystems, Bannockburn, IL). Lysates of two OSE and two FTE primary cultures and OVCA420 cell line were analyzed using standard Western blotting with CA125 antibody (Abcam, Cambridge, MA) and chemiluminescent signals were revealed using WesternBright ECL kit (Advansta, Menlo Park, CA).

### Statistic analysis of real-time PCR data

Non-parametric Kruskal Wallis test was performed to test differential expression between normal ovarian cells and various types of ovarian tumors for microRNA, target genes, and effector genes. The same test was adopted to test differential expression between normal FTE cells, STIC tumors, and fallopian tube stromal cells. If Kruskal Wallis test was found significant at the 0.05 levels, further multiple comparisons using Mann-Whitney U test were performed to test differential expression between various types of tumors against normal cells. The *P*-values of the Mann-Whitney test were corrected for multiple comparisons using the Bonferroni method. The correlation between microRNA, target genes, and effector genes was evaluated using Pearson correlation. In addition, two-way analysis of variance (ANOVA) was performed to assess the effects of various microRNA precursors on target and effector gene expression after transfection. Overall effects of each of the miRNA relative to control were compared using Dunnett’s test for multiple comparisons. Statistical analyses were performed using IBM SPSS-22 (IBM, Chicago, IL).

## Results and discussion

### Characterization of miR-200 pathway expression in ovarian and fallopian tube cells

Our previous gene expression profiling study of ovarian 3D cellular structures growing sequentially in Matrigel and then collagen I matrices which mimic inclusion cysts and invading tumors has identified concurrent elevated expression of miR-200 family members and effector protein CDH1, and decreased expression of transforming growth factor β (TGFβ) pathway genes in the cancer 3D cultures compared to the 3D cultures derived from normal OSE cells [26]. Suppression of CDH1 expression in ovarian cancer cells disrupted inclusion cyst formation and collective movement demonstrated in the cancer 3D cultures [26]. Identification of miR-200 targets is the key to understand the function of these miRNAs in ovarian cancer. E-cadherin transcriptional repressors *ZEB1* and *ZEB2* are two of the targets of miR-200 family and miR-205 [27]. Burk et al. have also identified *TGFβ* as another potential target of the miR-200 family [28]. Aigner et al. have shown that the transcription factor ZEB1 regulates besides E-cadherin other effector genes that are key determinants of epithelial cell phenotype [30]. *TGFβ*, the other miR-200 target, suppresses epithelial cell polarity by activating TGFβ receptors to phosphorylate the polarity complex protein Par6, which results in ubiquitination and degradation of Rho GTPase family member RhoA and the loss of tight junctions [31]. The members of the Par polarity complex interact with Lethal giant larvae (Lgl), a member of the basolateral Scribble complex and control apicobasal polarity, cell proliferation, migration and epithelial structure during tumor development [32]. Hence, the net results of miR-200 overexpression will be down-regulation of the target genes *ZEB1*, *ZEB2*, and *TGFβ*, with the corresponding up-regulation of multiple epithelial markers and more differentiated phenotype (MET) of the cells.

To evaluate expression of the miR-200 family and the associated pathway in ovarian cancer, we performed a large-scale quantitative real-time reverse transcription-polymerase chain reaction (qRT-PCRs) study with RNA from a panel of enriched ovarian cells from either primary cultures or laser-capture microdissection. These included 15 normal OSE preparations derived from five primary scrapes and microdissection from 10 archived frozen normal ovarian tissues, and epithelial tumor cells microdissected from three benign tumors, six borderline tumors, 14 nonserous malignant tumors (six mucinous, one clear cell, and seven endometrioid), 26 serous malignant tumors, 11 ovarian cancer cell lines, and six stromal preparations microdissected from ovarian tumor tissues. In this study, we also explored the potential involvement of miR-200 pathway in the hypothesized fallopian tube precursor lesions of high-grade serous tumors and hence have also collected four primary cultures of normal fallopian tube epithelial (FTE) cells derived from distal fallopian tube scrapes, epithelial tumor cells and stromal cells microdissected from three cases of frozen STIC tissues. qRT-PCRs were performed to determine the expression levels of the five members of the miR-200 family and miR-205, another miRNA involved in MET [27]; the target genes *ZEB1*, *ZEB2*, *TGFβ1*, and *TGFβ2*; and a panel of potential effector genes downstream of the ZEB1 transcriptional repressor selected from the article by Aigner et al. [30]. These included cadherins, claudins, *LLGL2* and tumor epithelial marker *EpCAM*. We have also included in the effector gene list two well-known ovarian tumor markers, *Muc16* (CA125) and *HE4*.

**Fig. 1:**
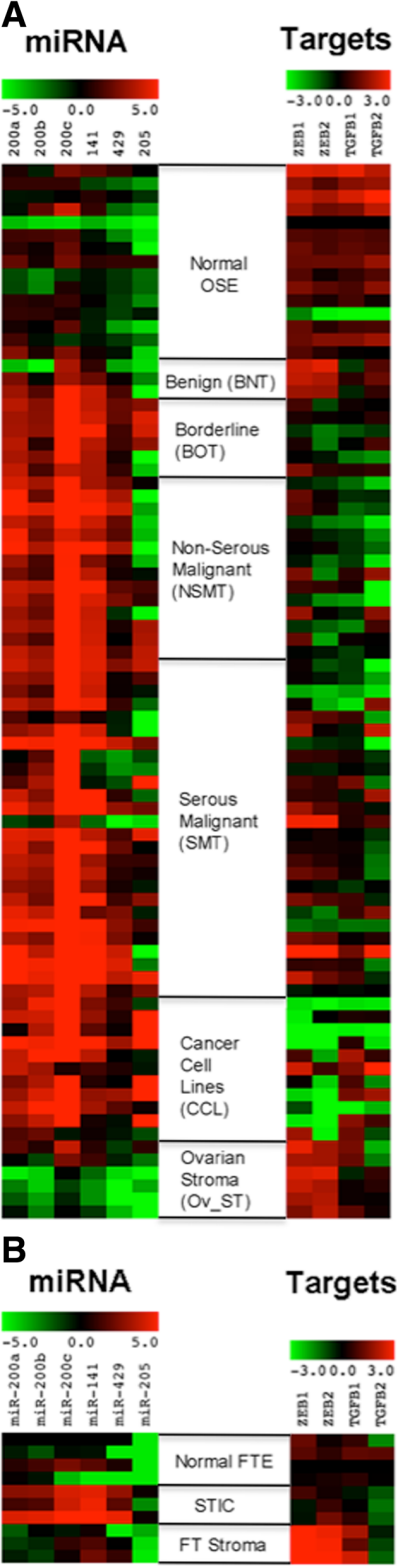
Heat maps showing the expression of miR-200 family and miR-205 and target genes in **a** ovarian and **b** fallopian tube cells

The heat maps in Fig. 1 serve as a visual presentation of the qRT-PCR results for miRNA and target genes. Unlike miR-205, the miR-200 family members showed in general upregulated expression in ovarian tumors and cancer cell lines when compared with normal OSE cells (Fig. 1a). Non-parametric Kruskal Wallis analyses of the qRT-PCR data showed that there were significant differences among groups in the miRNA expression (Table 1). Multiple comparisons using Mann-Whitney U test showed that miR-200 family members were all significantly overexpressed in the borderline (BOT), serous (SMT) and non-serous malignant tumors (NSMT), and cancer cell lines (CCL) compared with normal OSE cells. MiR-141 was the only RNA that was also significantly overexpressed in benign tumors (BNT) (*P* = 0.030). In our analysis, miR-205 was overexpressed only in SMT and CCL when compared with normal OSE cells. Albeit not significant in multiple comparisons, miR-200 expression appeared to be more downregulated in the ovarian stroma (OV_ST) than in OSE cells, in consistence with the notion that miR-200 are for epithelial differentiation. For the fallopian tube cells, there was an apparent increased expression of miR-200 in the STIC tumor cells than normal FTE cells (Fig. 1b), although multiple comparisons did not show significance because of the small sample size (Table 1).

**Table 1:** Differential expression of miR-200 and miR-205 in ovarian and fallopian tube cells

	Ovarian cells							Fallopian tube cells		
MicroRNA	Overall *P*-value	BNT	BOT	NSMT	SMT	CCL	Ov_ST	Overall *p*-value	STIC	FT_ST
		Difference in medians against normal cells (*P*-value)							Difference in medians against normal cells (*P*-value)	
MiR-200a	<0.0005^†^	1.807 (1.000)	2.839 (0.003*)	3.206 (0.001*)	3.081 (0.001*)	2.440 (0.002*)	−1.612 (0.672)	0.018^†^	2.613 (0.114)	−1.227 (0.114)
MiR-200b	<0.0005^†^	−0.160 (1.000)	2.537 (0.006*)	2.544 (0.001*)	3.060 (0.001*)	4.051 (0.001*)	−2.797 (0.396)	0.050^†^	2.970 (0.114)	−0.572 (1.000)
MiR-200c	<0.0005^†^	2.280 (0.060)	4.231 (0.004*)	4.516 (0.001*)	5.729 (0.001*)	5.919 (0.006*)	−1.120 (1.000)	0.055	4.331 (0.114)	0.450 (1.000)
MiR-141	<0.0005^†^	2.315 (0.030*)	3.978 (0.003*)	4.335 (0.001*)	4.412 (0.001*)	2.738 (0.001*)	−1.310 (1.000)	0.034^†^	4.883 (0.114)	1.702 (0.458)
MiR-429	<0.0005^†^	1.241 (1.000)	2.295 (0.005*)	2.825 (0.001*)	2.090 (0.004*)	1.664 (0.004*)	−2.897 (0.270)	0.047^†^	5.204 (0.114)	2.370 (1.000)
MiR-205	0.001^†^	−1.622 (1.000)	5.396 (0.792)	0.912 (1.000)	2.725 (0.030*)	8.156 (0.002*)	−1.664 (1.000)	0.021^†^	4.861 (0.114)	0.280 (0.458)

*BNT* Benign tumors, *BOT* Borderline tumors, *NSMT* Non-Serous malignant tumors, *SMT* Serous malignant tumors, *CCL* Cancer cell lines, *Ov_ST* Ovarian stroma, *STIC* Serous tubal intraepithelial carcinoma, *FT_ST* Fallopian tube stroma

^†^significant at the 0.05 level by Kruskal Wallis test

*significant at the 0.05 level by Mann-Whitney test (corrected using the Bonferroni method for multiple comparisons)

For the target genes, *ZEB1* was significantly underexpressed only in CCL (*P* = 0.048) and overexpressed in Ov_ST (*P* = 0.048) when compared with normal OSE cells (Fig. 1 and Table 2). On the other hand, *ZEB2* was broadly underexpressed in BOT (*P* = 0.048), NSMT (*P* = 0.002) and CCL (*P* = 0.001), but was overexpressed in Ov_ST (*P* = 0.012) when compared with normal OSE cells. *TGFβ1* was significantly underexpressed in BOT (*P* = 0.036), NSMT (*P* = 0.001) and SMT (*P* = 0.006); *TGFβ2* was underexpressed in NSMT (*P* = 0.006) and SMT (*P* = 0.012) when compared with normal OSE cells. Similar to the miRNA results, the target genes were not significantly different between normal FTE cells and STIC and FT stroma (FT_ST) on multiple comparisons, even though overall *P*-values were significant for *ZEB1*, *ZEB2* and *TGFβ1*.

**Table 2:** Differential expression of target genes in ovarian and fallopian tube cells

	Ovarian cells							Fallopian tube cells		
Target genes	Overall *P*-value	BNT	BOT	NSMT	SMT	CCL	Ov_ST	Overall *p*-value	STIC	FT_ST
		Difference in medians against normal cells (*P*-value)							Difference in medians against normal cells (*P*-value)	
ZEB1	<0.0005^†^	1.065 (0.336)	−0.880 (0.330)	−1.075 (0.156)	−0.602 (0.684)	−3.691 (0.048*)	0.925 (0.048*)	0.034^†^	−0.770 (0.458)	2.594 (0.114)
ZEB2	<0.0005^†^	1.167 (0.102)	−1.418 (0.048*)	−1.426 (0.002*)	−0.496 (0.516)	−4.083 (0.001*)	1.176 (0.012*)	0.034^†^	0.409 (0.458)	2.679 (0.114)
TGFB1	0.001^†^	−1.426 (0.234)	−1.009 (0.036*)	−1.669 (0.001*)	−0.902 (0.006*)	−0.129 (1.000)	−0.389 (1.000)	0.043^†^	−0.523 (0.800)	1.409 (0.114)
TGFB2	0.014^†^	−0.031 (1.000)	−0.718 (0.216)	−2.039 (0.006*)	−1.522 (0.012*)	−1.869 (0.282)	−0.819 (0.174)	0.352	−1.088 (0.800)	−0.812 (0.800)

^†^significant at the 0.05 level by Kruskal Wallis test

*significant at the 0.05 level by Mann-Whitney test (corrected using the Bonferroni method for multiple comparisons)

We also performed correlation analyses to determine the expression relationship within the miRNA group and between miRNA and target genes in all samples (Additional file 1: Table S1). MiR-200 family members showed very high expression correlation with each other (Pearson correlation coefficients r range between 0.666 and 0.889). MiR-205 showed modest correlations with miR-200b (*r* = 0.52) and miR-200c (*r* = 0.491). MiR-200b, miR-200c and miR-205 showed significant negative correlation with the expression of target genes *ZEB1* and *ZEB2*, but not with that of *TGFβ1* and *TGFβ2*, suggesting that the miRNA regulated MET primarily through *ZEB1* and *ZEB2*. However, the expression of *ZEB1* showed strong correlations with *ZEB2*, *TGFβ1* and *TGFβ2*. There was also significant correlation between *TGFβ1* and *TGFβ2* expression (Additional file 1: Table S1).

**Fig. 2:**
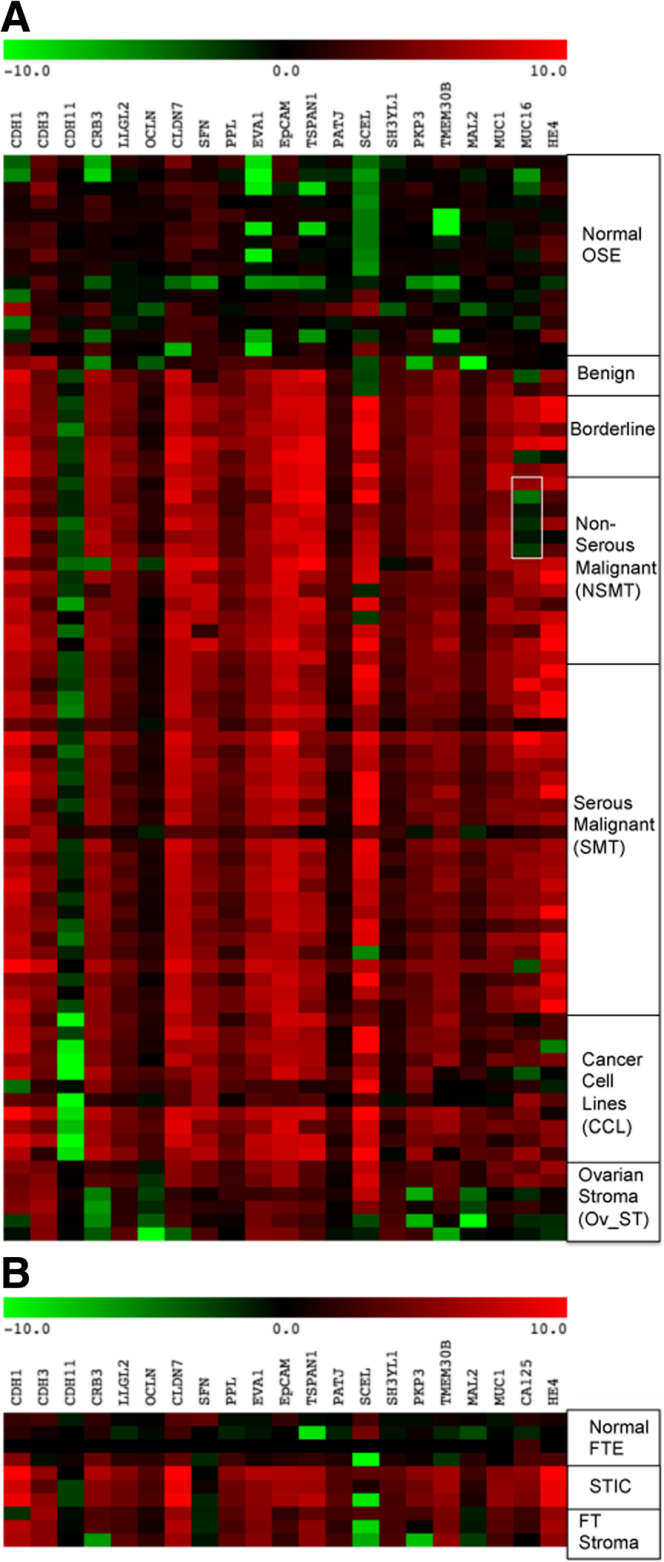
Heat maps showing the expression of effector genes in **a** ovarian and **b** fallopian tube cells. A *box* was drawn in the MUC16 lane to show the expression levels of mucinous ovarian tumors within the Non-serous malignant tumor samples

For the 21 effector genes that we measured (Fig. 2 and Table 3), all five categories (BNT, BOT, NSMT, SMT, and CCL) of ovarian tumor cells showed significantly elevated levels of *CDH1*, *PPL, EVA1, TSPAN1, SH3YL1*, and *TMEM30B* expression than normal OSE cells. In addition, *CRB3, LLGL2, CLDN7, SFN, EpCAM, PATJ, SCEL, PKP3, MAL2* and *MUC1* were elevated in BOT, NSMT, SMT, and CCL. In contrast, the expression of *CDH11* was negatively associated with these four tumor cell categories, consistent with the finding that this cadherin was associated with TGFβ production and EMT [33]. It was noted that the stromal cells extracted from tumor samples also expressed significant levels of *CDH3, LLGL2, EVA1, EpCAM, TSPAN1, SCEL*, and *SH3YL1*, suggesting that the tumor stroma also exhibited changes during the tumorigenesis process. Correlation analyses (Additional file 1: Table S2) showed that the expression of all miR-200 family was closely correlated. The expression of *CRB3, CLDN7, EpCAM*, and *PKP3* was strongly associated with the expression of all miR-200 family members, whereas the expression of *OCLN* and *MAL2* was highly correlated with miR-200a and miR-200b only. A core of epithelial effector genes including *CDH1, CRB3, LLGL2, CLDN7, PPL, EVA1, EpCAM, TSPAN1*, and *MUC1* had very high correlation coefficients (*r* ≥ 0.6) with each other and apparently were co-regulated in the samples.

**Table 3:** Differential expression of effector genes in ovarian and fallopian tube cells

	Ovarian cells							Fallopian tube cells		
Effector genes	Overall *P*-value	BNT	BOT	NSMT	SMT	CCL	OV_ST	Overall *P*-value	STIC	FT_ST
		Difference in medians against normal cells (*P*-value)							Difference in medians against normal cells (*P*-value)	
CDH1	<0.0005^†^	6.872 (0.030*)	7.070 (0.004*)	6.819 (0.001*)	6.479 (0.001*)	6.592 (0.002*)	3.552 (0.672)	0.050^†^	7.546 (0.458)	4.940 (0.114)
CDH3	<0.0005^†^	1.395 (0.102)	1.911 (0.006*)	1.370 (0.024*)	2.019 (0.001*)	1.120 (0.216)	2.323 (0.007*)	0.038^†^	5.370 (0.458)	5.112 (0.114)
CDH11	<0.0005^†^	−0.917 (1.000)	−2.679 (0.003*)	−2.953 (0.001*)	−2.444 (0.001*)	−9.627 (0.001*)	−0.198 (1.000)	0.445	−2.481 (0.458)	−0.272 (0.800)
CRB3	<0.0005^†^	4.786 (1.000)	5.302 (0.003*)	5.178 (0.001*)	4.652 (0.001*)	4.884 (0.001*)	−5.034 (1.000)	0.057	3.731 (0.458)	1.485 (0.114)
LLGL2	<0.0005^†^	3.633 (0.102)	4.472 (0.003*)	4.538 (0.001*)	2.882 (0.001*)	3.221 (0.001*)	1.654 (0.007*)	0.018^†^	4.495 (0.458)	2.745 (0.114)
OCLN	<0.0005^†^	1.231 (1.000)	1.014 (0.006*)	0.829 (0.096)	0.838 (0.003*)	1.590 (0.012*)	−2.429 (0.010*)	0.034^†^	3.096 (0.458)	1.215 (0.114)
CLDN7	<0.0005^†^	4.978 (0.984)	5.689 (0.003*)	5.336 (0.001*)	5.203 (0.001*)	4.896 (0.001*)	0.398 (1.000)	0.018^†^	6.995 (0.458)	3.327 (0.114)
SFN	<0.0005^†^	0.266 (1.000)	4.168 (0.003*)	4.638 (0.001*)	3.022 (0.001*)	4.412 (0.001*)	0.231 (1.000)	0.445	−0.184 (1.000)	−1.170 (0.800)
PPL	<0.0005^†^	3.186 (0.012*)	4.017 (0.003*)	2.941 (0.001*)	2.600 (0.001*)	3.099 (0.001*)	1.322 (1.000)	0.018^†^	4.336 (0.114)	3.684 (0.114)
EVA1	<0.0005^†^	11.177 (0.012*)	11.728 (0.003*)	11.459 (0.001*)	11.927 (0.001*)	11.146 (0.001*)	10.105 (0.003*)	0.018^†^	5.809 (0.114)	5.374 (0.114)
EpCAM	<0.0005^†^	6.759 (0.060)	7.272 (0.003*)	7.383 (0.001*)	6.692 (0.001*)	6.810 (0.001*)	3.405 (0.003*)	0.026^†^	5.720 (0.114)	1.208 (0.228)
TSPAN1	<0.0005^†^	7.922 (0.012*)	9.214 (0.003*)	8.662 (0.001*)	6.263 (0.001*)	7.030 (0.001*)	3.565 (0.003*)	0.018^†^	6.783 (0.114)	5.594 (0.114)
PATJ	<0.0005^†^	1.605 (0.234)	2.106 (0.006*)	1.841 (0.001*)	1.210 (0.001*)	1.202 (0.006*)	0.233 (0.924)	0.018^†^	3.229 (0.114)	2.521 (0.114)
SCEL	<0.0005^†^	1.685 (1.000)	15.068 (0.003*)	12.210 (0.001*)	13.000 (0.001*)	13.054 (0.001*)	10.820 (0.036*)	0.943	−0.230 (1.000)	−8.010 (1.000)
SH3YL1	<0.0005^†^	2.466 (0.012*)	2.778 (0.003*)	2.285 (0.001*)	1.681 (0.001*)	0.840 (0.024*)	2.126 (0.003*)	0.030^†^	3.300 (0.114)	3.064 (0.114)
PKP3	<0.0005^†^	2.905 (1.000)	4.467 (0.003*)	4.135 (0.001*)	3.401 (0.001*)	4.066 (0.001*)	0.935 (1.000)	0.057	4.463 (0.114)	0.151 (1.000)
TMEM30B	<0.0005^†^	5.936 (0.012*)	6.352 (0.003*)	6.083 (0.001*)	5.493 (0.001*)	5.362 (0.004*)	3.300 (0.084)	0.024^†^	7.019 (0.114)	5.539 (0.114)
MAL2	<0.0005^†^	1.066 (1.000)	1.895 (0.003*)	1.577 (0.001*)	1.753 (0.001*)	1.507 (0.006*)	−2.284 (1.000)	0.095	1.447 (0.458)	−1.279 (0.800)
MUC1	<0.0005^†^	3.849 (0.234)	5.906 (0.003*)	5.480 (0.001*)	4.651 (0.001*)	2.027 (0.036*)	1.138 (0.270)	0.018^†^	5.808 (0.114)	3.414 (0.114)
MUC16	<0.0005^†^	0.370 (1.000)	6.820 (0.048*)	5.030 (0.246)	5.531 (0.001*)	3.535 (0.006*)	0.630 (1.000)	0.055	3.044 (0.114)	0.130 (1.000)
HE4	<0.0005^†^	1.890 (1.000)	6.100 (0.108)	5.400 (0.006*)	5.681 (0.001*)	1.359 (1.000)	−1.090 (1.000)	0.018^†^	8.767 (0.114)	5.247 (0.114)

^†^significant at the 0.05 level by Kruskal Wallis test

*significant at the 0.05 level by Mann-Whitney test (corrected using the Bonferroni method for multiple comparisons)

It is also of interest that the two clinical ovarian cancer markers, *MUC16* and *HE4*, were not highly expressed in all tumor cell categories. *HE4* was significantly elevated only in NSMT and SMT, and *MUC16* was significantly elevated only in BOT, SMT, and CCL, but not in NSMT (Table 3). More significantly, mucinous malignant tumors (marked by a box in Fig. 2) showed a markedly reduced level of *MUC16* expression, which was significantly different from the expression in endometrioid (*P* = 0.024) and high-grade serous (*P* = 0.012) malignant tumors (Additional file 1: Table S3). Tumor type-dependent expression differences of HE4 [34] and CA125 [35] have been described previously. There was a high correlation (*r* = 0.646) in the expression between these two biomarkers. *HE4* also had additional expression correlations with *CLDN7* (*r* = 0.619) and *MUC1* (*r* = 0.605, Additional file 1: Table S2).

For the fallopian tube cells, there were no significant differences in effector gene expression despite significant overall *P*-values (Table 3). Based on the fact that the heat maps for the expression of miRNAs, target genes and effector genes in fallopian tube cells were similar to the heat maps for the ovarian cells (Figs. 1 and 2), it is very likely that the lack of significant differences between STIC tumor cells and normal FTE cells was due to small sample sizes.

### Stimulation of epithelial marker expression in normal OSE and FTE cells and specific induction of CA125 expression in FTE cells by miR-200 family

**Fig. 3:**
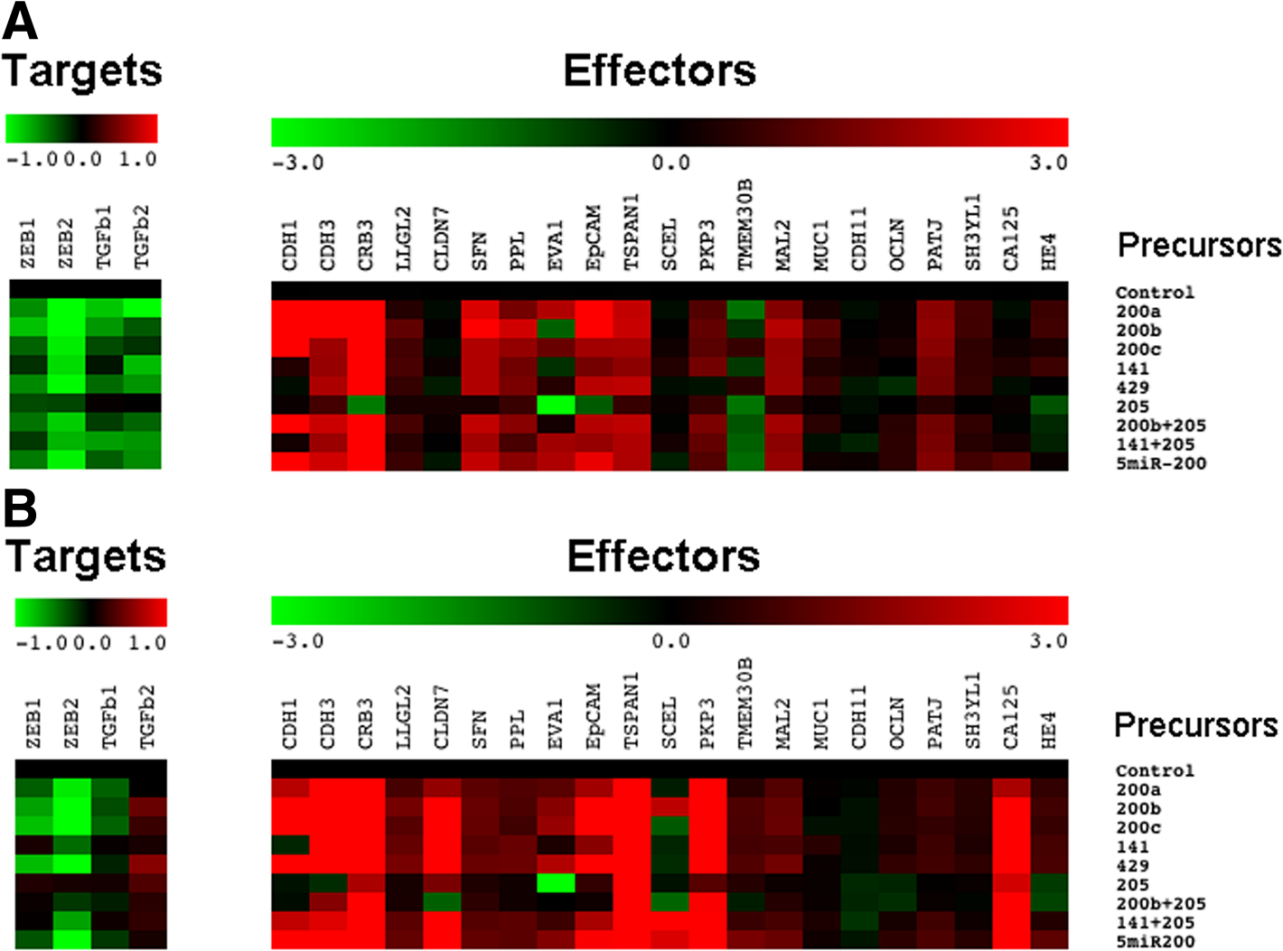
Heat maps showing the expression of target and effector genes after transfection of control and different combinations of miR-200 and miR-205 precursors into **a** normal OSE and **b** normal FTE cells

To explore further the relationships between microRNA, target genes, and effector genes, individuals and combinations of synthetic miR-200 precursors were transfected into normal OSE and FTE cells and the expression of target genes and effector genes was analyzed from the transfected cells using qRT-PCR. The heat maps in Fig. 3 and two-way ANOVA analysis in Additional file 1: Tables S4 and S5 show the responses of target genes and effector genes upon introduction of miR-200 and miR-205 precursor molecules. First, miR-205 was found not very effective in inducing changes in the expression of target genes (*P* = 0.935 and 0.986) and effector genes (*P* = 0.941 and 0.763) in OSE and FTE cells, respectively, suggesting that miR-205 does not play any important role in the miR-200 pathway in OSE and FTE cells. The miR-200 family precursor molecules, however, were effective in suppressing expression of all target genes in OSE cells and *ZEB1* and *ZEB2* in FTE (Additional file 1: Table S4), and induce the expression of effector genes in OSE and FTE cells (Additional file 1: Table S5). This is consistent with the correlation analysis results (Additional file 1: Table S1), which showed that the expression of miR-200 was more correlated with *ZEB1* and *ZEB2* expression than with *TGFβ1* and *TGFβ2* expression. The miR-200 family members did not contribute equally to affect the expression of target genes and effector genes. While the introduction of miR-200 precursors into both normal OSE and FTE cells stimulated the expression of cadherins *CDH1* and *CDH3*, and effector genes like *CRB3, EpCAM, TSPAN1*, and *PKP3* that had high correlation coefficients with miR-200 expression, there were striking differences of responses in some effector gene expression from these two normal cell types (Additional file 1: Table S5). *CLDN7*, the expression of which was highly correlated with miR-200 in correlation analyses (Additional file 1: Table S2), was only induced in FTE cells (Additional file 1: Table S5). Another interesting finding was the expression of *MUC16*, which in correlation analysis was not related to any miR-200 or any other epithelial marker expression except *HE4*, was found to be induced only in FTE cells (Fig. 3 and Additional file 1: Table S5). In contrast, *HE4* expression was not induced at all by miR-200 introduction in both normal cell types.

**Fig. 4:**
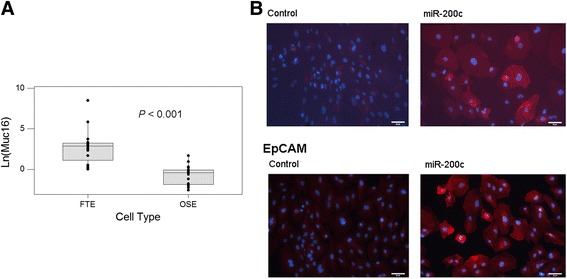
Induction of effector gene expression in FTE cells after miR-200 transfection. **a** *Box plot* to show the differential expression of *Muc16* mRNA in FTE cells and OSE cells after the transfection. **b** Immunostaining of CA125 and EpCAM proteins after the transfection of either nontarget control RNA or miR-200 precursor RNA into FTE cells. Positive protein targets were pseudo-colored in *red*, and nuclear staining was in *blue*

We repeated the miRNA precursor transfections with different preparations of normal OSE and FTE cells and the results (Fig. 4a) confirmed the specific *MUC16* mRNA expression mediated by miR-200 precursor molecules in FTE cells and not in OSE cells. Furthermore, immunostaining of the normal FTE cells confirmed the increased expression of EpCAM and CA125 proteins in the FTE cells after transfection with miR-200 precursors (Fig. 4b). A Western blot analysis performed with lysates derived from two OSE and two FTE primary cultures (Additional file 1: Fig. S1) showed that there was negligible basal expression of CA125 in both OSE and FTE cells.

## Conclusions

Our study demonstrated activation of the miR-200 pathway in ovarian tumors, ovarian cancer cell lines, and likely in the fallopian tube STIC cells as well. Activation of miR-200 pathway in ovarian and fallopian tube cancer cells confirms the previous findings of suppression of TGFβ pathway [36] and epithelial characteristics of ovarian cancer cells when compared with OSE and likely FTE [8, 37–39]. Manipulations of epithelial marker E-cadherin expression in OSE and ovarian cancer cells have been shown to affect tumor formation [40, 41] and tumor invasion via collective cell movement [26]. A recent characterization of a mouse model of high-grade serous ovarian cancer originating in the fallopian tube stroma showed epithelialization of the stromal cancer cells to support tumorigenesis [42]. The miR-200 pathway may play an important role in regulating MET and promoting epithelial-like oncogenesis in OSE and/or FTE cells. Our second finding that normal FTE cells have greater propensity than OSE cells to express CA125 in response to miR-200 expression is intriguing. Since high-grade serous ovarian tumors express higher levels of CA125 than the nonserous mucinous ovarian tumors [43, 44], our finding is consistent with the notions that at least some of the high-grade serous ovarian tumors originate in the fallopian tube and nonserous mucinous tumors might develop from the ovarian surface epithelium or cortical inclusion cysts. Since our expression analysis did not reveal any significant correlation between miR-200 expression and *MUC16* expression, it will be of great interest to determine the underlying mechanism that regulates *MUC16* expression by miR-200 in FTE cells. This warrants further investigation and, hopefully, will provide important information about the etiologies of histologic subtypes of ovarian cancer.

## Additional file

Additional file 1:
**Fig. S1.** Western blot analysis to show negligible expression of CA125 (MUC16) in OSE and FTE primary cultures. **Table S1.** Correlation analyses for the relationship between expression levels of miR-200 family and miR-205 and target genes. **Table S2.** Correlation analyses for the expression relationship between miR-200 family and miR-205 and effector genes. **Table S3.** Differential expression of effector gene MUC16 in different subtypes of ovarian tumors. **Table S4.** Effects of microRNA on target gene expression in OSE and FTE cells. **Table S5.** Effects of microRNA on effector gene expression in OSE and FTE (PDF 445 kb)
