# Supplementary material for: Characterization of MicroRNA-200 pathway in ovarian cancer and serous intraepithelial carcinoma of fallopian tube
Source: BMC Cancer. 2017 Jun 17;17:422. doi: 10.1186/s12885-017-3417-z (PMC5473983; doi:10.1186/s12885-017-3417-z)
Supplement: Additional file 1: — Fig. S1. Western blot analysis to show negligible expression of CA125 (MUC16) in OSE and FTE primary cultures. Table S1. Correlation analyses for the relationship between expression levels of miR-200 family and miR-205 and target genes. Table S2. Correlation analyses for the expression relationship between miR-200 family and miR-205 and effector genes. Table S3. Differential expression of effector gene MUC16 in different subtypes of ovarian tumors. Table S4. Effects of microRNA on target gene expression in OSE and FTE cells. Table S5. Effects of microRNA on effector gene expression in OSE and FTE (PDF 445 kb) [file 12885_2017_3417_MOESM1_ESM.pdf]

Supplemental Information

One Supplemental Figure and
Five Supplemental Tables

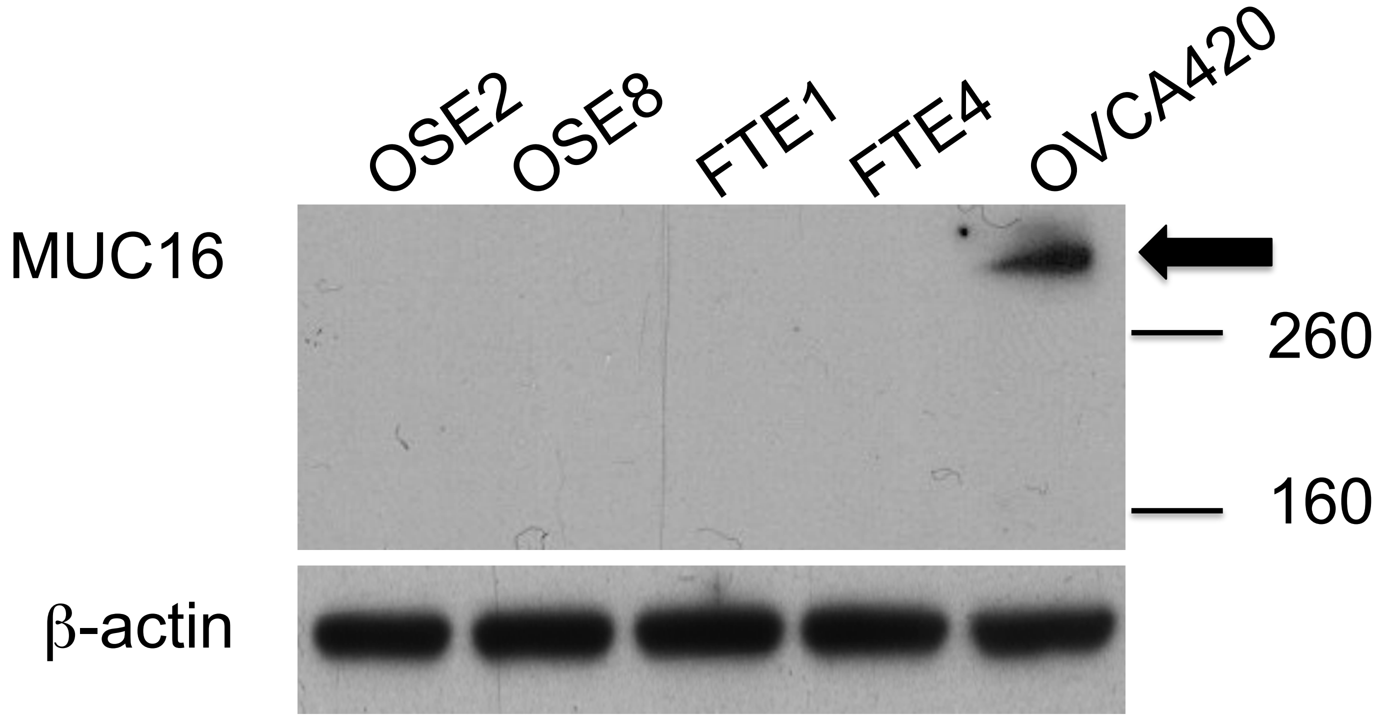

Figure S1. Western blot analysis to show negligible expression of CA125 (MUC16) in OSE and FTE primary cultures. OVCA420 cancer cell line was used as the reference for MUC16 expression (block arrow). β-actin was used as loading control.

Table S1. Correlation analyses for the relationship between expression levels of miR-200 family and miR-205 and target genes.

		miR-200a	miR-200b	miR-200c	miR-141	miR-429	miR-205	ZEB1	ZEB2	TGFB1	TGFB2
miR-200a	Pearson Correlation	1	.863**	.726**	.866**	.889**	.329**	-.268*	-.246*	-.178	-.242*
	Sig. (2-tailed)		.000	.000	.000	.000	.003	.015	.027	.111	.029
miR-200b	Pearson Correlation	.863**	1	.834**	.711**	.757**	.520**	-.474**	-.464**	-.208	-.297**
	Sig. (2-tailed)	.000		.000	.000	.000	.000	.000	.000	.062	.007
miR-200c	Pearson Correlation	.726**	.834**	1	.750**	.666**	.491**	-.409**	-.369**	-.281*	-.307**
	Sig. (2-tailed)	.000	.000		.000	.000	.000	.000	.001	.011	.005
miR-141	Pearson Correlation	.866**	.711**	.750**	1	.886**	.330**	-.204	-.180	-.235*	-.211
	Sig. (2-tailed)	.000	.000	.000		.000	.003	.067	.107	.035	.058
miR-429	Pearson Correlation	.889**	.757**	.666**	.886**	1	.397**	-.294**	-.295**	-.167	-.244*
	Sig. (2-tailed)	.000	.000	.000	.000		.000	.008	.007	.136	.028
miR-205	Pearson Correlation	.329**	.520**	.491**	.330**	.397**	1	-.529**	-.483**	-.012	-.254*
	Sig. (2-tailed)	.003	.000	.000	.003	.000		.000	.000	.919	.022
ZEB1	Pearson Correlation	-.268*	-.474**	-.409**	-.204	-.294**	-.529**	1	.709**	.507**	.537**
	Sig. (2-tailed)	.015	.000	.000	.067	.008	.000		.000	.000	.000
ZEB2	Pearson Correlation	-.246*	-.464**	-.369**	-.180	-.295**	-.483**	.709**	1	.356**	.301**
	Sig. (2-tailed)	.027	.000	.001	.107	.007	.000	.000		.001	.006
TGFB1	Pearson Correlation	-.178	-.208	-.281*	-.235*	-.167	-.012	.507**	.356**	1	.582**
	Sig. (2-tailed)	.111	.062	.011	.035	.136	.919	.000	.001		.000
TGFB2	Pearson Correlation	-.242*	-.297**	-.307**	-.211	-.244*	-.254*	.537**	.301**	.582**	1
	Sig. (2-tailed)	.029	.007	.005	.058	.028	.022	.000	.006	.000	

N=81 for all. **. Correlation is significant at the 0.01 level (2-tailed). *. Correlation is significant at the 0.05 level (2-tailed).

Correlation coefficients that are ≥ 4 are highlighted in red, whereas the correlation coefficients that are ≤ -4.0 are highlighted in blue.

Table S2. Correlation analyses for the expression relationship between miR-200 family and miR-205 and effector genes.

		miR-200a	miR-200b	miR-200c	miR-141	miR-429	miR-205	CDH1	CDH3	CDH11	CRB3	LLGL2	OCLN
miR-200a	Pearson Correlation	1	.863**	.726**	.866**	.889**	.329**	.505**	.075	-.281*	.686**	.612**	.528**
	Sig. (2-tailed)		.000	.000	.000	.000	.003	.000	.508	.011	.000	.000	.000
	N	81	81	81	81	81	81	81	81	81	81	81	81
miR-200b	Pearson Correlation	.863**	1	.834**	.711**	.757**	.520**	.537**	.127	-.517**	.728**	.518**	.625**
	Sig. (2-tailed)	.000		.000	.000	.000	.000	.000	.257	.000	.000	.000	.000
	N	81	81	81	81	81	81	81	81	81	81	81	81
miR-200c	Pearson Correlation	.726**	.834**	1	.750**	.666**	.491**	.728**	.433**	-.435**	.666**	.573**	.483**
	Sig. (2-tailed)	.000	.000		.000	.000	.000	.000	.000	.000	.000	.000	.000
	N	81	81	81	81	81	81	81	81	81	81	81	81
miR-141	Pearson Correlation	.866**	.711**	.750**	1	.886**	.330**	.569**	.218	-.188	.607**	.620**	.451**
	Sig. (2-tailed)	.000	.000	.000		.000	.003	.000	.051	.094	.000	.000	.000
	N	81	81	81	81	81	81	81	81	81	81	81	81
miR-429	Pearson Correlation	.889**	.757**	.666**	.886**	1	.397**	.464**	.037	-.254*	.591**	.537**	.550**
	Sig. (2-tailed)	.000	.000	.000	.000		.000	.000	.744	.022	.000	.000	.000
	N	81	81	81	81	81	81	81	81	81	81	81	81
miR-205	Pearson Correlation	.329**	.520**	.491**	.330**	.397**	1	.305**	.162	-.437**	.343**	.162	.325**
	Sig. (2-tailed)	.003	.000	.000	.003	.000		.006	.147	.000	.002	.149	.003
	N	81	81	81	81	81	81	81	81	81	81	81	81
CDH1	Pearson Correlation	.505**	.537**	.728**	.569**	.464**	.305**	1	.473**	-.331**	.731**	.690**	.414**
	Sig. (2-tailed)	.000	.000	.000	.000	.000	.006		.000	.003	.000	.000	.000
	N	81	81	81	81	81	81	81	81	81	81	81	81
CDH3	Pearson Correlation	.075	.127	.433**	.218	.037	.162	.473**	1	-.021	.120	.348**	.041
	Sig. (2-tailed)	.508	.257	.000	.051	.744	.147	.000		.854	.287	.001	.718
	N	81	81	81	81	81	81	81	81	81	81	81	81
CDH11	Pearson Correlation	-.281*	-.517**	-.435**	-.188	-.254*	-.437**	-.331**	-.021	1	-.334**	-.290**	-.291**
	Sig. (2-tailed)	.011	.000	.000	.094	.022	.000	.003	.854		.002	.009	.008
	N	81	81	81	81	81	81	81	81	81	81	81	81
CRB3	Pearson Correlation	.686**	.728**	.666**	.607**	.591**	.343**	.731**	.120	-.334**	1	.567**	.620**
	Sig. (2-tailed)	.000	.000	.000	.000	.000	.002	.000	.287	.002		.000	.000
	N	81	81	81	81	81	81	81	81	81	81	81	81
LLGL2	Pearson Correlation	.612**	.518**	.573**	.620**	.537**	.162	.690**	.348**	-.290**	.567**	1	.334**
	Sig. (2-tailed)	.000	.000	.000	.000	.000	.149	.000	.001	.009	.000		.002
	N	81	81	81	81	81	81	81	81	81	81	81	81
OCLN	Pearson Correlation	.528**	.625**	.483**	.451**	.550**	.325**	.414**	.041	-.291**	.620**	.334**	1
	Sig. (2-tailed)	.000	.000	.000	.000	.000	.003	.000	.718	.008	.000	.002	
	N	81	81	81	81	81	81	81	81	81	81	81	81
CLDN7	Pearson Correlation	.643**	.668**	.727**	.656**	.590**	.381**	.712**	.435**	-.288**	.659**	.727**	.577**
	Sig. (2-tailed)	.000	.000	.000	.000	.000	.000	.000	.000	.009	.000	.000	.000
	N	81	81	81	81	81	81	81	81	81	81	81	81
SFN	Pearson Correlation	.575**	.625**	.585**	.505**	.471**	.307**	.599**	.199	-.411**	.634**	.732**	.388**
	Sig. (2-tailed)	.000	.000	.000	.000	.000	.005	.000	.075	.000	.000	.000	.000
	N	81	81	81	81	81	81	81	81	81	81	81	81
PPL	Pearson Correlation	.452**	.470**	.603**	.554**	.436**	.362**	.720**	.400**	-.227*	.612**	.774**	.474**
	Sig. (2-tailed)	.000	.000	.000	.000	.000	.001	.000	.000	.041	.000	.000	.000
	N	81	81	81	81	81	81	81	81	81	81	81	81
EVA1	Pearson Correlation	.439**	.463**	.622**	.488**	.329**	.271*	.737**	.414**	-.328**	.646**	.697**	.163
	Sig. (2-tailed)	.000	.000	.000	.000	.003	.015	.000	.000	.003	.000	.000	.145
	N	81	81	81	81	81	81	81	81	81	81	81	81
EpCAM	Pearson Correlation	.662**	.651**	.744**	.644**	.530**	.286**	.780**	.412**	-.345**	.680**	.865**	.374**
	Sig. (2-tailed)	.000	.000	.000	.000	.000	.010	.000	.000	.002	.000	.000	.001
	N	81	81	81	81	81	81	81	81	81	81	81	81
TSPAN1	Pearson Correlation	.561**	.496**	.609**	.533**	.481**	.244*	.730**	.256*	-.382**	.634**	.795**	.273*
	Sig. (2-tailed)	.000	.000	.000	.000	.000	.028	.000	.021	.000	.000	.000	.014
	N	81	81	81	81	81	81	81	81	81	81	81	81
PATJ	Pearson Correlation	.461**	.460**	.483**	.518**	.439**	.173	.679**	.290**	-.238*	.555**	.710**	.350**
	Sig. (2-tailed)	.000	.000	.000	.000	.000	.121	.000	.009	.032	.000	.000	.001
	N	81	81	81	81	81	81	81	81	81	81	81	81
SCEL	Pearson Correlation	.505**	.544**	.558**	.433**	.414**	.423**	.598**	.244*	-.314**	.602**	.565**	.288**
	Sig. (2-tailed)	.000	.000	.000	.000	.000	.000	.000	.028	.004	.000	.000	.009
	N	81	81	81	81	81	81	81	81	81	81	81	81
SH3YL1	Pearson Correlation	.247*	.159	.397**	.392**	.175	.024	.532**	.577**	.028	.389**	.618**	.128
	Sig. (2-tailed)	.027	.157	.000	.000	.118	.833	.000	.000	.806	.000	.000	.256
	N	81	81	81	81	81	81	81	81	81	81	81	81
PKP3	Pearson Correlation	.744**	.759**	.604**	.606**	.629**	.383**	.598**	.085	-.352**	.806**	.660**	.577**
	Sig. (2-tailed)	.000	.000	.000	.000	.000	.000	.000	.450	.001	.000	.000	.000
	N	81	81	81	81	81	81	81	81	81	81	81	81
TMEM30B	Pearson Correlation	.550**	.543**	.737**	.627**	.535**	.350**	.708**	.459**	-.207	.552**	.668**	.398**
	Sig. (2-tailed)	.000	.000	.000	.000	.000	.001	.000	.000	.063	.000	.000	.000
	N	81	81	81	81	81	81	81	81	81	81	81	81
MAL2	Pearson Correlation	.630**	.656**	.513**	.520**	.601**	.325**	.454**	-.008	-.250*	.693**	.471**	.618**
	Sig. (2-tailed)	.000	.000	.000	.000	.000	.003	.000	.945	.024	.000	.000	.000
	N	81	81	81	81	81	81	81	81	81	81	81	81
MUC1	Pearson Correlation	.638**	.478**	.600**	.693**	.588**	.205	.651**	.245*	-.012	.581**	.779**	.386**
	Sig. (2-tailed)	.000	.000	.000	.000	.000	.067	.000	.028	.914	.000	.000	.000
	N	81	81	81	81	81	81	81	81	81	81	81	81
MUC16	Pearson Correlation	.278*	.360**	.446**	.352**	.236*	.497**	.441**	.177	-.140	.411**	.409**	.151
	Sig. (2-tailed)	.012	.001	.000	.001	.034	.000	.000	.113	.213	.000	.000	.179
	N	81	81	81	81	81	81	81	81	81	81	81	81
HE4	Pearson Correlation	.475**	.426**	.530**	.522**	.402**	.367**	.488**	.307**	.008	.450**	.419**	.308**
	Sig. (2-tailed)	.000	.000	.000	.000	.000	.001	.000	.005	.943	.000	.000	.005
	N	81	81	81	81	81	81	81	81	81	81	81	81

		CLDN7	SFN	PPL	EVA1	EpCAM	TSPAN1	PATJ	SCEL	SH3YL1	PKP3	TMEM30B	MAL2	MUC1	MUC16	HE4
miR-200a	Pearson Correlation	.643**	.575**	.452**	.439**	.662**	.561**	.461**	.505**	.247*	.744**	.550**	.630**	.638**	.278*	.475**
	Sig. (2-tailed)	.000	.000	.000	.000	.000	.000	.000	.000	.027	.000	.000	.000	.000	.012	.000
	N	81	81	81	81	81	81	81	81	81	81	81	81	81	81	81
miR-200b	Pearson Correlation	.668**	.625**	.470**	.463**	.651**	.496**	.460**	.544**	.159	.759**	.543**	.656**	.478**	.360**	.426**
	Sig. (2-tailed)	.000	.000	.000	.000	.000	.000	.000	.000	.157	.000	.000	.000	.000	.001	.000
	N	81	81	81	81	81	81	81	81	81	81	81	81	81	81	81
miR-200c	Pearson Correlation	.727**	.585**	.603**	.622**	.744**	.609**	.483**	.558**	.397**	.604**	.737**	.513**	.600**	.446**	.530**
	Sig. (2-tailed)	.000	.000	.000	.000	.000	.000	.000	.000	.000	.000	.000	.000	.000	.000	.000
	N	81	81	81	81	81	81	81	81	81	81	81	81	81	81	81
miR-141	Pearson Correlation	.656**	.505**	.554**	.488**	.644**	.533**	.518**	.433**	.392**	.606**	.627**	.520**	.693**	.352**	.522**
	Sig. (2-tailed)	.000	.000	.000	.000	.000	.000	.000	.000	.000	.000	.000	.000	.000	.001	.000
	N	81	81	81	81	81	81	81	81	81	81	81	81	81	81	81
miR-429	Pearson Correlation	.590**	.471**	.436**	.329**	.530**	.481**	.439**	.414**	.175	.629**	.535**	.601**	.588**	.236*	.402**
	Sig. (2-tailed)	.000	.000	.000	.003	.000	.000	.000	.000	.118	.000	.000	.000	.000	.034	.000
	N	81	81	81	81	81	81	81	81	81	81	81	81	81	81	81
miR-205	Pearson Correlation	.381**	.307**	.362**	.271*	.286**	.244*	.173	.423**	.024	.383**	.350**	.325**	.205	.497**	.367**
	Sig. (2-tailed)	.000	.005	.001	.015	.010	.028	.121	.000	.833	.000	.001	.003	.067	.000	.001
	N	81	81	81	81	81	81	81	81	81	81	81	81	81	81	81
CDH1	Pearson Correlation	.712**	.599**	.720**	.737**	.780**	.730**	.679**	.598**	.532**	.598**	.708**	.454**	.651**	.441**	.488**
	Sig. (2-tailed)	.000	.000	.000	.000	.000	.000	.000	.000	.000	.000	.000	.000	.000	.000	.000
	N	81	81	81	81	81	81	81	81	81	81	81	81	81	81	81
CDH3	Pearson Correlation	.435**	.199	.400**	.414**	.412**	.256*	.290**	.244*	.577**	.085	.459**	-.008	.245*	.177	.307**
	Sig. (2-tailed)	.000	.075	.000	.000	.000	.021	.009	.028	.000	.450	.000	.945	.028	.113	.005
	N	81	81	81	81	81	81	81	81	81	81	81	81	81	81	81
CDH11	Pearson Correlation	-.288**	-.411**	-.227*	-.328**	-.345**	-.382**	-.238*	-.314**	.028	-.352**	-.207	-.250*	-.012	-.140	.008
	Sig. (2-tailed)	.009	.000	.041	.003	.002	.000	.032	.004	.806	.001	.063	.024	.914	.213	.943
	N	81	81	81	81	81	81	81	81	81	81	81	81	81	81	81
CRB3	Pearson Correlation	.659**	.634**	.612**	.646**	.680**	.634**	.555**	.602**	.389**	.806**	.552**	.693**	.581**	.411**	.450**
	Sig. (2-tailed)	.000	.000	.000	.000	.000	.000	.000	.000	.000	.000	.000	.000	.000	.000	.000
	N	81	81	81	81	81	81	81	81	81	81	81	81	81	81	81
LLGL2	Pearson Correlation	.727**	.732**	.774**	.697**	.865**	.795**	.710**	.565**	.618**	.660**	.668**	.471**	.779**	.409**	.419**
	Sig. (2-tailed)	.000	.000	.000	.000	.000	.000	.000	.000	.000	.000	.000	.000	.000	.000	.000
	N	81	81	81	81	81	81	81	81	81	81	81	81	81	81	81
OCLN	Pearson Correlation	.577**	.388**	.474**	.163	.374**	.273*	.350**	.288**	.128	.577**	.398**	.618**	.386**	.151	.308**
	Sig. (2-tailed)	.000	.000	.000	.145	.001	.014	.001	.009	.256	.000	.000	.000	.000	.179	.005
	N	81	81	81	81	81	81	81	81	81	81	81	81	81	81	81
CLDN7	Pearson Correlation	1	.691**	.713**	.697**	.862**	.698**	.629**	.556**	.403**	.678**	.796**	.503**	.729**	.464**	.619**
	Sig. (2-tailed)		.000	.000	.000	.000	.000	.000	.000	.000	.000	.000	.000	.000	.000	.000
	N	81	81	81	81	81	81	81	81	81	81	81	81	81	81	81
SFN	Pearson Correlation	.691**	1	.678**	.648**	.781**	.691**	.540**	.550**	.364**	.740**	.584**	.510**	.577**	.450**	.364**
	Sig. (2-tailed)	.000		.000	.000	.000	.000	.000	.000	.001	.000	.000	.000	.000	.000	.001
	N	81	81	81	81	81	81	81	81	81	81	81	81	81	81	81
PPL	Pearson Correlation	.713**	.678**	1	.661**	.755**	.679**	.696**	.606**	.577**	.596**	.663**	.476**	.688**	.566**	.453**
	Sig. (2-tailed)	.000	.000		.000	.000	.000	.000	.000	.000	.000	.000	.000	.000	.000	.000
	N	81	81	81	81	81	81	81	81	81	81	81	81	81	81	81
EVA1	Pearson Correlation	.697**	.648**	.661**	1	.819**	.818**	.558**	.646**	.559**	.516**	.687**	.291**	.627**	.511**	.426**
	Sig. (2-tailed)	.000	.000	.000		.000	.000	.000	.000	.000	.000	.000	.009	.000	.000	.000
	N	81	81	81	81	81	81	81	81	81	81	81	81	81	81	81
EpCAM	Pearson Correlation	.862**	.781**	.755**	.819**	1	.867**	.651**	.661**	.566**	.735**	.799**	.504**	.773**	.506**	.560**
	Sig. (2-tailed)	.000	.000	.000	.000		.000	.000	.000	.000	.000	.000	.000	.000	.000	.000
	N	81	81	81	81	81	81	81	81	81	81	81	81	81	81	81
TSPAN1	Pearson Correlation	.698**	.691**	.679**	.818**	.867**	1	.603**	.658**	.484**	.605**	.775**	.393**	.716**	.386**	.376**
	Sig. (2-tailed)	.000	.000	.000	.000	.000		.000	.000	.000	.000	.000	.000	.000	.000	.001
	N	81	81	81	81	81	81	81	81	81	81	81	81	81	81	81
PATJ	Pearson Correlation	.629**	.540**	.696**	.558**	.651**	.603**	1	.468**	.355**	.529**	.594**	.336**	.563**	.401**	.417**
	Sig. (2-tailed)	.000	.000	.000	.000	.000	.000		.000	.001	.000	.000	.002	.000	.000	.000
	N	81	81	81	81	81	81	81	81	81	81	81	81	81	81	81
SCEL	Pearson Correlation	.556**	.550**	.606**	.646**	.661**	.658**	.468**	1	.334**	.579**	.585**	.426**	.536**	.492**	.351**
	Sig. (2-tailed)	.000	.000	.000	.000	.000	.000	.000		.002	.000	.000	.000	.000	.000	.001
	N	81	81	81	81	81	81	81	81	81	81	81	81	81	81	81
SH3YL1	Pearson Correlation	.403**	.364**	.577**	.559**	.566**	.484**	.355**	.334**	1	.292**	.494**	.159	.563**	.300**	.309**
	Sig. (2-tailed)	.000	.001	.000	.000	.000	.000	.001	.002		.008	.000	.156	.000	.007	.005
	N	81	81	81	81	81	81	81	81	81	81	81	81	81	81	81
PKP3	Pearson Correlation	.678**	.740**	.596**	.516**	.735**	.605**	.529**	.579**	.292**	1	.513**	.839**	.617**	.414**	.457**
	Sig. (2-tailed)	.000	.000	.000	.000	.000	.000	.000	.000	.008		.000	.000	.000	.000	.000
	N	81	81	81	81	81	81	81	81	81	81	81	81	81	81	81
TMEM30B	Pearson Correlation	.796**	.584**	.663**	.687**	.799**	.775**	.594**	.585**	.494**	.513**	1	.335**	.731**	.390**	.501**
	Sig. (2-tailed)	.000	.000	.000	.000	.000	.000	.000	.000	.000	.000		.002	.000	.000	.000
	N	81	81	81	81	81	81	81	81	81	81	81	81	81	81	81
MAL2	Pearson Correlation	.503**	.510**	.476**	.291**	.504**	.393**	.336**	.426**	.159	.839**	.335**	1	.520**	.357**	.441**
	Sig. (2-tailed)	.000	.000	.000	.009	.000	.000	.002	.000	.156	.000	.002		.000	.001	.000
	N	81	81	81	81	81	81	81	81	81	81	81	81	81	81	81
MUC1	Pearson Correlation	.729**	.577**	.688**	.627**	.773**	.716**	.563**	.536**	.563**	.617**	.731**	.520**	1	.548**	.605**
	Sig. (2-tailed)	.000	.000	.000	.000	.000	.000	.000	.000	.000	.000	.000	.000		.000	.000
	N	81	81	81	81	81	81	81	81	81	81	81	81	81	81	81
MUC16	Pearson Correlation	.464**	.450**	.566**	.511**	.506**	.386**	.401**	.492**	.300**	.414**	.390**	.357**	.548**	1	.646**
	Sig. (2-tailed)	.000	.000	.000	.000	.000	.000	.000	.000	.007	.000	.000	.001	.000		.000
	N	81	81	81	81	81	81	81	81	81	81	81	81	81	81	81
HE4	Pearson Correlation	.619**	.364**	.453**	.426**	.560**	.376**	.417**	.351**	.309**	.457**	.501**	.441**	.605**	.646**	1
	Sig. (2-tailed)	.000	.001	.000	.000	.000	.001	.000	.001	.005	.000	.000	.000	.000	.000	
	N	81	81	81	81	81	81	81	81	81	81	81	81	81	81	81

**. Correlation is significant at the 0.01 level (2-tailed). *. Correlation is significant at the 0.05 level (2-tailed).

Correlations that are ≥ 0.6 are highlighted in red.

Table S3: Differential expression of effector gene *MUC16* in different subtypes of ovarian tumors

Effector gene	Ovarian cancer cells			
	Overall *P*-value	Clear cell	Endometrioid	Serous
		Difference in medians against Mucinous cancer cells (*P*-value)		
MUC16	<0.016†	7.979 (0.858)	7.819 (0.024*)	6.911 (0.012*)

† significant at the 0.05 level by Kruskal Wallis test

* significant at the 0.05 level by Mann-Whitney test (corrected using the Bonferroni method for multiple comparisons)

Table S4: Effects of microRNA on target gene expression in OSE and FTE cells

	MicroRNA								
	miR-200a	miR-200b	miR-200c	miR-141	miR-429	miR-205	miR-200b + miR-205	miR-141 + miR-205	Five miR-200 members
Overall effect (OSE)	-1.010	-0.782	-0.452	-0.468	-0.629	-0.133	-0.472	-0.534	-0.587
P-value† (OSE)	<0.001*	<0.001*	0.029*	0.022*	0.001*	0.935	0.021*	0.007*	0.003*
Overall effect (FTE)	-0.419	-0.512	-0.578	-0.047	-0.454	0.156	-0.031	-0.088	-0.360
P-value† (FTE)	0.340	0.163	0.090	1.000	0.261	0.986	1.000	1.000	0.499

Target gene	Estimated marginal mean expression	*P*-value‡
ZEB1 (OSE)	-0.389	<0.001*
ZEB2 (OSE)	-0.854	<0.001*
TGFB1 (OSE)	-0.338	<0.001*
TGFB2 (OSE)	-0.449	<0.001*
ZEB1 (FTE)	-0.252	0.016*
ZEB2 (FTE)	-0.752	<0.001*
TGFB1 (FTE)	-0.135	0.183
TGFB2 (FTE)	0.204	0.048*

† p-value of Dunnett's test to compare each microRNA versus Controls (two-sided)

‡ p-value for testing mean expressions not equal to zero (two-sided)

* significant at the 0.05 level

Table S5: Effects of microRNA on effector gene expression in OSE and FTE

	MicroRNA								
	miR-200a	miR-200b	miR-200c	miR-141	miR-429	miR-205	miR-200b + miR-205	miR-141 + miR-205	Five miR-200 members
Overall effect (OSE)	1.493	1.684	1.351	0.935	0.835	-0.286	1.276	0.918	1.404
P-value† (OSE)	<0.001*	<0.001*	<0.001*	0.023*	0.055	0.941	0.001*	0.027*	<0.001*
Overall effect (FTE)	1.588	2.440	2.203	1.798	2.540	0.412	0.772	1.945	2.519
P-value† (FTE)	<0.001*	<0.001*	<0.001*	<0.001*	<0.001*	0.763	0.127	<0.001*	<0.001*

Effector gene	Estimated marginal mean expression (OSE)	*P*-value‡ (OSE)	Estimated marginal mean expression (FTE)	*P*-value‡ (FTE)
CDH1	2.973	<0.001*	2.565	<0.001*
CDH3	1.981	<0.001*	2.779	<0.001*
CRB3	3.781	<0.001*	5.337	<0.001*
LLGL2	0.608	0.059	0.875	0.011*
CLDN7	-0.016	0.960	3.061	<0.001*
SFN	1.856	<0.001*	0.894	0.009*
PPL	1.303	<0.001*	0.774	0.024*
EVA1	0.015	0.963	0.614	0.072
EpCAM	1.774	<0.001*	1.946	<0.001*
TSPAN1	1.744	<0.001*	4.470	<0.001*
SCEL	0.027	0.933	0.411	0.227

PKP3	0.847	0.009*	3.040	<0.001*
TMEM30B	-0.624	0.053	0.627	0.066
MAL2	1.468	<0.001*	0.830	0.015*
MUC1	0.362	0.259	0.136	0.689
CDH11	-0.057	0.859	-0.249	0.464
OCLN	0.158	0.622	0.212	0.532
PTAJ	1.237	<0.001*	0.549	0.107
SH3YL1	0.503	0.118	0.309	0.363
MUC16	0.163	0.611	4.496	<0.001*
HE4	0.081	0.800	0.380	0.264

† p-values of Dunnett's test to compare each microRNA versus Controls (two-sided)

‡ p-values for testing mean expressions not equal to zero (two-sided)

* significant at the 0.05 level
